# Locating sex-specific evidence on clinical questions in MEDLINE: a search filter for use on OvidSP™

**DOI:** 10.1186/1471-2288-9-25

**Published:** 2009-04-14

**Authors:** Clara J Moerman, Rikie Deurenberg, Joke A Haafkens

**Affiliations:** 1 Department of General Practice, Division of Clinical Methods and Public Health, Academic Medical Center-University of Amsterdam, Meibergdreef 15, 1105 AZ Amsterdam, the Netherlands; 2 Dutch Institute for Healthcare Improvement CBO, Churchilllaan 11, 3527 GV Utrecht, the Netherlands

## Abstract

**Background:**

Many recently published clinical studies report sex-specific data. This information may help to improve clinical decision-making for both sexes, but it is not easily accessible in MEDLINE. The aim of this project was to develop and validate a search filter that would facilitate the retrieval of studies reporting high quality sex-specific data on clinical questions.

**Methods:**

A filter was developed by screening titles, abstracts and Medical Subject Headings (MeSH) in a set of 80 high quality and relevant papers, 75 of which were identified through a review of clinical guidelines and five through other means. The filter, for use on OvidSP™, consists of nine command lines for searching free text words in the title, abstract and MeSH of a paper. It was able to identify 74/80 (92.5%) of the articles from which it was derived. The filter was evaluated in a set of 622 recently published original studies on Alzheimer's disease and on asthma. It was validated against a reference of 98 studies from this set, which provided high quality, clinically relevant, sex-specific evidence. Recall and precision were used as performance measures.

**Results:**

The filter demonstrated 81/98 (83%) recall and 81/125 (65%) precision in retrieving relevant articles on Alzheimer's disease and on asthma. In comparison, only 30/98 (31%) recall would have been achieved if sex-specific MeSH terms only had been used.

**Conclusion:**

This sex-specific search filter performs well in retrieving relevant papers, while its precision rate is good. It performs better than a search with sex-specific MeSH. The filter can be useful to anyone seeking sex-specific clinical evidence (e.g., guideline organizations, researchers, medical educators, clinicians).

## Background

Research-based evidence is an important foundation for clinical decision making. In the past, women have often been underrepresented among participants in clinical research [1-3]. Since the 1990s, however, health research funding organizations have taken initiatives to redress this bias, and researchers have begun to pay more attention to the equitable inclusion of men and women in clinical research and the analysis of the data according to sex [4-11]. This has led to a new body of published research data on differences between and among men and women in the aetiology, diagnosis, treatment and prevention of diseases [12-18]. These differences may be rooted in the biological or physiological "sex" characteristics that define men and women or in their socially constructed "gender" roles, behaviours or activities [3,19]. For the sake of simplicity, in this paper we will use the term "sex-specific evidence" to refer to both sex and gender-related research-based data on differences between or among men and women.

One example of sex-specific evidence was reported by a study on malignant melanoma. The study found that men have a poorer life expectancy as compared to women, even when the melanoma is less thick [13]. Another example concerns the use of the AUDIT screening instrument for identifying alcohol problems. A systematic review revealed that the instrument was consistently less sensitive and more specific for women than for men when the recommended cut-off point of 8 was used. This led to the conclusion that for women a lower cut-off point (for instance 5) would be needed to identify these problems [18]. In order to provide optimal care to both men and women, it is essential that this new sex-specific evidence is easily accessible to anyone who is interested in evidence-based clinical practice (e.g., clinicians, guideline developers, health educators, researchers).

MEDLINE is one of the most widely consulted bibliographic databases for biomedical literature. In a previous project that investigated the uptake of sex-specific data in Dutch clinical guidelines [20], we discovered that finding sex-specific evidence in MEDLINE can be a difficult task. One problem is that searches for this type of evidence cannot be limited to a restricted number of journals, whereas sex-specific data are published in many different journals and not only in those focusing on gender medicine. MEDLINE offers a number of possibilities to facilitate searches for articles on sex-specific topics. Using the Check tags Male or Female is one possibility. However, these Check tags are assigned to an article if the concepts male or female are mentioned anywhere in the text. They will, therefore, retrieve all articles that mention either males (Check tag Male) or females (Check tag Female) and not only those providing information on differences among them. Another possibility is to use Medical Subject Headings, or MeSH, from the controlled vocabulary thesaurus. This thesaurus is composed by indexers of the National Library of Medicine (NLM) to describe the subject content of articles for MEDLINE. It contains several MeSH descriptors for sex-specific evidence (Table 1). Yet, it is not certain if these sex-specific MeSH capture all the relevant research reports. They may not be comprehensive enough, or they may be applied inconsistently by indexers. An example is the inconsistent indexing of studies evaluating sex as an effect modifier of the relationship under study [21,22]. For these reasons, searching MEDLINE for articles that contain sex-specific evidence can be frustrating. On the one hand searches may yield many irrelevant research reports. On the other hand it is not certain that all relevant reports are included in the search results.

**Table 1:** Medical Subject Headings (MeSH) considered relevant for locating sex-specific clinical evidence and their definitions.

MeSH term	Definition (Scope Note)
Sex factors	Maleness or femaleness as a constituent element or influence contributing to the production of a result. It may be applicable to the cause or effect of a circumstance. It is used with human or animal concepts but should be differentiated from SEX CHARACTERISTICS, anatomical or physiological manifestations of sex, and from SEX DISTRIBUTION, the number of males and females in given circumstances.

Sex characteristics	Those characteristics that distinguish one SEX from the other. The primary sex characteristics are the OVARIES and TESTES and their related hormones. Secondary sex characteristics are those which are masculine or feminine but not directly related to reproduction.

Sex distribution	The number of males and females in a given population. The distribution may refer to how many men or women or what proportion of either in the group. The population is usually patients with a specific disease but the concept is not restricted to humans and is not restricted to medicine.

Sex	The totality of characteristics of reproductive structure, functions, PHENOTYPE, and GENOTYPE, differentiating the MALE from the FEMALE organism.

Sex ratio	The number of males per 100 females.

Gender identity	Socially-constructed identity of male or female.
	NOTE: psychological; do not confuse with SEX CHARACTERISTICS (physiological); do not confuse with SEXUAL ORIENTATION see SEX BEHAVIOR: gender identity is knowing that one is male or female; sexual orientation is preferring heterosexual or homosexual behavior; no qualif.

Search filters can be useful tools to facilitate detection of specific information in MEDLINE. As part of a follow-up project to improve attention to sex-specific factors in guideline development [23], two major Dutch guideline development organizations asked us to develop a search filter that could improve access to high quality evidence with respect to clinical questions, for both women and men. To date, little empirical work has been done in this area [24,25]. One previously published filter contains some search terms for finding information on sex differences (see Table 2) [26]. We did not consider this filter suitable to our end, because it was developed to answer questions on women's health only. Moreover, the authors did not specify the criteria they had used for classifying the quality of the articles that had been identified by the filter. For that reason, we conducted a study that had the aim of developing and validating a search filter for locating relevant sex-specific evidence related to clinical questions in MEDLINE. This paper describes the results.

**Table 2:** Search filter for locating research papers on women's health developed by Montgomery and Sherif [26]

**Term**^**a**^
sex factors
women's health/
women's health services/
exp women/
sex characteristics/
sex distribution/
sex determination/
gender identity/
journal of the american medical women's association.jn.
women & health.jn.
womens health issues.jn.
health care for women international.jn.
gender$.tw.
women$.tw.
woman$.tw.
female$.tw.
girl$.tw.
mother$.tw.
widow$.tw.

^**a **^The extension .jn means journal, .tw means free text word. Terms without these extensions are MeSH. All terms should be used together in an OR (either) relationship.

## Methods

The filter was developed by researchers with ample experience in the retrieval of literature in biomedical databases and the development of search strategies.

Our goal was to develop a search filter that would facilitate the retrieval of high quality research describing sex-specific data on clinical questions related to conditions that can occur in both sexes. We excluded conditions that occur only in one sex, such as heavy menstrual bleeding, whereas literature on those conditions can be located simply by using disease-specific search terms (MeSH).

Jenkins has described a number of possible methodologies for filter development [27]. Given the absence of previous work on the selection of sex-specific search terms and the composition of a gold standard of studies for the evaluation and validation of those terms, we have followed Jenkins' recommendation to develop a so called "second generation filter". A characteristic of second generation filters is that search terms are subjectively derived and tested against a gold standard. A novelty of our approach was that we wanted to ensure that the papers that were selected for developing and validating the filter would also be considered as relevant by clinicians. To achieve this goal we composed two sets of published papers that had been critically reviewed for methodological soundness and clinical relevance by experts, prior to the study. The first set, that was used to develop the search filter, was selected from clinical guidelines that were developed by organizations that belong to the Appraisal of Guidelines for Research and Evaluation (AGREE) collaboration. These guideline organizations use the internationally developed AGREE instrument for testing the quality of the guidelines they produce [28]. Some of the AGREE quality criteria pertain to the selected evidence, which should be clearly evaluated for methodological soundness and clinical relevance. The second set of papers, that was used to validate the filter, was selected from a core set of leading clinical journals. The publication policy of these journals requires that published papers are carefully reviewed for level of clinical interest and methodological quality.

### Development of the filter

Our aim was to identify 80 papers. Firstly, we selected 56 guidelines from a larger set of clinical guidelines published by four guideline organizations: the Dutch College of General Practitioners (NHG), the Dutch Institute for Healthcare Improvement (CBO), the Scottish Intercollegiate Guidelines Network (SIGN) and the National Institute for Health and Clinical Excellence (NICE, United Kingdom). Each of these organizations use a similar evidence-based methodology for the critical appraisal of the quality and the clinical relevance of research reports, as well as the aforementioned AGREE instrument for evaluating the quality of the final guideline documents [29-31]. To be selected, a guideline should have been published in 2006 or 2007, provide an answer to clinical questions and deal with a condition that could occur in both men and women.

Secondly, these guidelines were screened for statements about men (boys), women (girls) or differences between them. Statements were included if they referred to any of the following topics: risk factors, the natural course of the disease, diagnostics (including disease manifestation and test performance), treatment or prognosis. Twenty-two of the 56 selected guidelines included such statements. Subsequently, for each of the included statements, we selected one reference to the underlying literature, based on the following criteria: the article must be written in English, published between 1996 and 2007, indexed in MEDLINE and contain an abstract. We excluded references to consensus papers or systematic reviews, as our aim was to identify papers on original studies.

Using this process we identified 75 research papers. Topics of these papers included: cancer (colon cancer), heart disease (chronic heart failure, familial hypercholesterolemia, secondary prevention after myocardial infarction, stable angina, stroke), other chronic disease (asthma in children and adults, Type 2 diabetes, osteoarthritis of the hip and knee, rheumatoid arthritis, thyroid disorders), infectious disease (hepatitis C, tuberculosis), mental health conditions (alcohol dependence, bipolar disease, dementia, attention-deficit hyperactivity disorders, eating disorders), neurologic disease (Parkinson) and others (enuresis nocturna). To complete our target of 80 papers we added five other papers to this set: two (on heart failure and Type 2 diabetes) were identified through the website of the US Agency for Healthcare Research and Quality http://www.ahrq.gov/research/womenh1.htm[32,33], and three through a recent report on sex differences in rheumatoid arthritis [34]. We checked if these papers were indexed with one of the sex-specific MeSH as listed in Table 1. This was the case for 41 of the 80 papers (51%).

To identify potentially relevant search terms by which the papers in this set could be located in MEDLINE, the citations were downloaded and the OvidSP™ interface was used to screen the title, abstract and the MeSH of the individual articles. This interface was selected because it is commonly available in medical institutions and guideline organizations [35]. Moreover, we assumed that the more often a word is used in the abstract, the more important the topic will be (frequency). Likewise, we also assumed that the more closely two words are put together in the abstract, the more likely it is that their meaning is connected (adjacency). In contrast to other interfaces, such as PubMed, OvidSP™ offers operators for searching for the adjacency and frequency of words. This was another reason for selecting this interface.

For each of the 80 papers we registered all the words referring to male or female (either children or adults), sex and gender. Secondly, we registered the various combinations and frequencies by which these words appeared in title, abstract and MeSH as well as how closely they were located together. RD chose four as the minimum criterion for frequency and eight as the maximum criterion for adjacency. This choice was based on her prior experience with filter development and some tests. Finally, these data were examined to identify common patterns of terms by which a substantial number of the articles could be located. This led to the formulation of the sex-specific search filter (SSS filter). (Table 3)

**Table 3:** Sex-specific search filter (SSS filter) for MEDLINE for use with the OvidSP™ interface

	**Search terms**^**a**^
#1	(gender$ or sex$).af.
#2	(boys or girls).tw.
#3	(women or men).ti.
#4	(male$1 or female$1).ti.
#5	(women or men).ab./freq=4
#6	(male$1 or female$1).ab./freq=4
#7	(women adj8 men).ab.
#8	(female$1 adj8 male$1).ab.
#9	or/1–8

	The filter can be combined with a disease or other topic by adding search commands for the disease or topic and combining these commands with the SSS filter by using the Boolean operator 'AND'.

	We recommend to do a search in the leading clinical journals on women's health in addition to a search with the SSS filter as described above. The following Ovid search terms for journals can be used: gender medicine.jn., journal of womens health.jn., journal of womens health & gender based medicine.jn. in combination with the disease or topic in question.

^a ^The affixes indicate the location in which a specific word is searched for: ab = abstract; af = all fields (the affix is used to cover a search in titles, abstracts and Medical Subject Headings (MeSH)); ti = title; tw = (text word in) title or abstract. The additional command freq 4 means in a frequency of four times or more. The command adj8 between two words means that the second word should occur within eight words of the first word. The order of the words does not count; the first word can precede the second or the second the first. Practically, the two words are parted by seven other words at the most.

The SSS filter consists of nine command lines to search for free text words in the fields containing information about the title, abstract and MeSH of individual articles. The first eight lines include one or more text words, followed by an affix, indicating the fields in which the words in question should be located (e.g., all fields (including title, abstract and MeSH) or a selection of those fields). In addition, lines #5 and #6 include a frequency operator. This operator is used to indicate the minimum frequency with which the terms male or female, or men or women, should appear in the abstract. Lines #7 and #8 include an adjacency operator. This operator is used to be able to identify statements in the abstract in which the terms women and men or male and female are used in combination with each other. Line #9 combines lines #1 to #8 by using the Boolean operator 'OR'.

The SSS filter was able to identify 74 of the 80 articles (92.5%) from the set it was derived from. Four of the six papers that could not be identified mentioned sex-specific information in the body of the text, but not in the fields that were searched by the filter (title, abstract and MeSH). The two other papers mentioned sex-specific information in the abstract, but the way in which this was phrased could not be recognized by the filter.

### Validation of the filter

To validate the SSS filter we composed a reference set of papers through a search in MEDLINE [36]. To be included in the reference set, a paper must report recent primary research on Alzheimer's disease or on asthma in humans, be published in core clinical journals and contain sex-specific evidence relevant to answer clinical questions. We chose asthma as a topic because the disease occurs in all age groups, including children. Alzheimer's disease was added as a random choice. We limited our search to core clinical journals because those journals are selected by the NLM as being of immediate interest to the practicing physician.

As a first step we searched all articles on Alzheimer's disease and on asthma that were published in core clinical journals in 2007 and 2008 and included in the MEDLINE database as of 13-06-2008. To this end we used the MeSH for the two diseases (exp Alzheimer Disease/or exp asthma/) and corresponding free text words in titles and abstracts (Alzheimer?.mp or asthma.mp). Only articles in the English language that contain an abstract were included. Studies involving animals were excluded. In order to obtain reports of original studies, papers of clinical conferences or consensus development conferences, congresses, (practice) guidelines, meta-analyses, reviews, and technical reports were also excluded, using MEDLINE's categorization by publication type.

As a second step we made a selection within this set by singling out the papers containing potentially relevant sex-specific information. To this end we screened the titles and the abstracts of the identified papers for the words (wo)man, (wo)men, (fe)male, widow(er), boy(s), girl(s), mother, father, sex or gender and MeSH including the words sex or gender. The papers that met these criteria were downloaded and their content was critically reviewed using the following criteria: a paper obtained a positive score if it reported data on men or women (or boys or girls) or the differences between them; if it evaluated the role of sex/gender as an independent variable or predictor for the outcome of the study or if it evaluated the role of sex/gender as an effect modifier for the relationship under study (see Table 4 for the criteria). We first evaluated information in the title and the abstract sections of the paper. If titles and abstracts did not provide sufficient information to decide whether relevant sex-specific evidence was present or not, we also evaluated information in the methods and results sections of the paper. The initial assessment of the papers was performed by the first author (CM). In case of doubt the papers were discussed with a second author (JH) until agreement was reached. The papers with a positive score formed the reference set.

**Table 4:** Criteria for the presence of sex-specific evidence relevant to clinical questions in primary research papers

Statements in title or abstract concerning:
Men (boys)
Women (girls)
Differences between the sexes
(Evaluation of) Independent effect of sex/gender on the relationship under study
(Evaluation of) Sex/gender as predictor for the outcome of the study
(Evaluation of) Sex/gender as an effect modifier of the relationship under study
**When the above-mentioned statements in title or abstract are absent:**
Screening full text for statements regarding methods of analysis:
Comparison between the sexes
Comparison between groups of a single sex
Stratification by sex or subgroup analysis by sex
Evaluation of sex/gender as effect modifier
Evaluation of the independent effect of sex/gender on the relationship under study
Evaluation of sex/gender as an independent predictor for the study outcome
*Statements regarding methods must be followed by a report about the outcome of this particular analysis*.
Screening full text for statements regarding reporting of results:
Presentation of separate risk estimates for men (boys) and women (girls)
Presentation estimating the differences between (groups within) the sexes
Outcome of a subgroup analysis by sex

We evaluated the performance of the SSS filter on the set of original papers on Alzheimer's disease and on asthma. We used recall (the number of papers containing relevant sex-specific evidence on clinical questions retrieved by the SSS filter as a proportion of the total number of papers in the reference set) and precision (the number of papers containing relevant sex-specific evidence on clinical questions retrieved as a proportion of the total number of papers retrieved) as measures of performance [27].

As a second test, we compared the performance of our filter to that of two other filters: a combination of previously selected sex-specific MeSH (Table 1) (SS MeSH filter) and the previously published filter for retrieving information on women's health (M&S filter) (see Table 2[26]). To this end we applied the other two filters to the set of original papers on Alzheimer's disease and on asthma and compared the yields with those of the SSS filter. For this comparison we did not search in women's health journals, as is recommended by the M&S filter, because those journals are not represented in the set of core clinical journals, which was a selection criterion for the reference set.

## Results

We found 662 articles reporting primary research on Alzheimer's disease and on asthma. One hundred and sixty-four of these papers were critically reviewed, as a first screening suggested that they might contain potentially relevant sex-specific information. This review found that 98 papers reported relevant sex-specific evidence on clinical questions. These 98 papers formed the reference set (see Table 5 for examples of sex-specific phrases found during the assessment). They were published in 24 different journals.

**Table 5:** Examples of primary research papers meeting the criteria as defined in Table 4

Title	"Endogenous sex hormones as risk factors for dementia in elderly men and women"
	"Interactions between breast-feeding, specific parental atopy, and sex on development of asthma and atopy"
	"Dynamic hyperinflation with bronchoconstriction: differences between obese and nonobese women with asthma"
Abstract	"to determine the relationship of aeroallergen sensitization to age, sex, ethnicity"
	"we sought to study the interrelations of allergy markers and FEV(1) in relation to asthma and sex"
	"the results were similar among both men and women"
	"among white patients, adherence was significantly lower for women when compared with men."
	"sex and age modified the patterns of concordance of high IgE levels, (...) with the greatest overlap in male children and the lowest in male adults"
	"independent risk factors for death were age, male gender (...)"
	"there were minor differences in the impact of parental disease (...) between boys and girls; interactions between parental disease and the child's allergic sensitization or gender were not statistically significant"

Text	Methods: "To examine whether effect modification was present by age cohort, sex, or level of cognitive test score at first examination, three stratified Cox proportional hazards models were calculated (...)." The results for men and women were presented separately in a table.
	
	Results: "To see whether the relation of conscientiousness to AD varied by sex, we repeated the original model with a term for the interaction of conscientiousness and sex. There was no evidence of an interaction of conscientiousness with sex in this model or with age or education in separate subsequent analyses (data not shown)."
	
	Results: "This analysis was repeated in people with depression; with the limitation due to loss of power, in this subgroup gender emerged as an important risk factor, with men having a threefold increase in mortality rate compared to women (HR, 3.30; 95% CI, 1.53 to 10.35) (...)."

The SSS filter retrieved 125 of the 662 primary research papers (57 on Alzheimer's disease, 68 on asthma). Eighty-one of the retrieved papers contained relevant sex-specific information. Sixty-one of these papers could be identified by screening the title and abstract. For the other 20 articles the relevance became clear only after reading the body of the text. The recall of the filter was 83% and the precision 65% (Table 6). The recall for Alzheimer's disease was high (97%) but, as a trade-off, the precision was considerably lower (49%); for asthma these figures were respectively 77% and 78%.

**Table 6:** Performance of three filters for locating articles^a ^reporting sex-specific evidence: SSS, SS MeSH and M&S

Disease	Filter	Number of articles			Recall	Precision
		Reference set	Retrieved by the filter	Retrieved and reporting sex-specific evidence		
		(X)	(Y)	(Z)	(Z/X)	(Z/Y)

Alzheimer's disease	SSS	29	57	28	97%	49%
	SS MeSH^b^	29	15	10	34%	67%
	M&S^c^	29	39	16	55%	41%
						
Asthma	SSS	69	68	53	77%	78%
	SS MeSH	69	23	20	29%	87%
	M&S	69	79	57	83%	72%
						
Total	SSS	98	125	81	83%	65%
	SS MeSH	98	38	30	31%	79%
	M&S	98	118	73	74%	62%

^a ^The filters were applied to articles on original studies of Alzheimer's disease and of asthma in humans, with an abstract, written in English, published in core clinical journals in 2007 and 2008, and included in MEDLINE as of June 13 2008. Thus, 662 articles on original studies were identified, including 219 articles on Alzheimer's disease and 443 articles on asthma.

^b ^Sex-specific MeSH (SS MeSH) include the following MeSH combined with the operator 'OR': sex factors, sex characteristics, sex distribution, sex, sex ratio and gender identity.

^c ^Modification of the filter as designed by Montgomery and Sherif [26]. Included were the MeSH terms referring to sex differences, gender issues and women's health, free text words referring to women and the text word gender (see Table 2); excluded was a search in women's health journals.

A search using SS MeSH (the sex-specific MeSH as listed in Table 1 combined with the operator 'OR') captured 38 of the 662 primary research papers. The precision of this filter (79%) was high as compared to the SSS filter. Its recall, however, was low (31%) (Table 6).

The M&S filter was able to retrieve 118 papers with a recall of 74% and a precision of 62% (Table 6). The SSS filter had a slightly higher recall and precision. Upon closer inspection, the SSS filter was clearly more sensitive than the M&S filter for Alzheimer's disease, but for asthma the performance of both filters was in a comparable range. We did not execute a formal test to evaluate the differences in performance between the two filters.

## Discussion

In this project we developed a search filter to facilitate the detection of sex-specific research evidence relevant to clinical questions in MEDLINE. This filter has been developed for use on OvidSP™. It contains free text words for searching the title, abstract and MeSH of publications. Overall, the filter was able to locate 83% of all the publications reporting sex-specific data, with a precision rate of 65%. Thus, it was somewhat less successful in filtering out non-relevant information. This can be explained by the fact that there is always a trade-off between recall and precision: as a rule of thumb, high recall is accompanied by considerably lower precision and vice versa. A sensitive search filter is particularly useful for detecting information that is not readily available. As MEDLINE only contains a relatively small number of papers reporting sex-specific evidence on clinical questions, it is relevant to use a sensitive search filter, like ours, for detecting this information. Even though the filter also achieved a good precision rate, future studies may focus on potential ways to improve this.

We intended to identify search terms that were able to identify high quality sex-specific evidence on clinical questions. There is no gold standard for this type of evidence. We believe, however, that we have taken rigorous measures to make sure that the data sets that were used to develop and evaluate the filter contained high quality and relevant research evidence. To develop the filter we used research reports with sex-specific data that had been reviewed according to international standards for quality appraisal and considered relevant to clinical practice by guideline developers. To test the performance of the filter, we chose papers from a set of clinical journals that were selected by the National Library of Medicine on the basis of their quality and clinical relevance. Moreover, in the further selection of the reference studies we used rigorous and transparent quality criteria. In our opinion, the size of our gold standard (n = 98) was acceptable for establishing the filter's recall, given the fact that the minimum size of gold standards in filter development is a hundred [35].

A limitation of our study is the generalizability of the findings. The SSS filter was developed and tested on the basis of clinical studies with designs that are able to provide strong evidence for or against a causal effect, such as randomized controlled trials. It has been argued that such studies may not always provide sufficient information about the socio-demographic and sex-specific characteristics of the research population [37] and that relevant evidence may also be found in non-interventional designs such as observational or qualitative studies [38]. These study types were underrepresented in our validation set. It should also be noted that the selection process of the SSS filter's search terms included subjective choices. That is, the decision to search for a frequency of four and an adjacency of eight was based on previous experience with filter development. A different frequency (e.g. 3 or 5) or adjacency might have resulted in a different recall.

Further research is required in various directions. As a start, the filter should be tested against a gold standard that is larger in size than ours and on studies about other diseases. Furthermore, research is needed to investigate whether the SSS filter would perform differently in the retrieval of studies with non-experimental designs. Further work is also needed to create and validate a version of the SSS filter for other bibliographic databases and search interfaces.

Validation for the search interface PubMed would be a first important step, because it is widely used in the medical community. One obstacle is that some of the operators which are an important efficiency factor in the SSS filter, such as adjacency and frequency, are not available in the current PubMed interface (Jan 2009). It would be useful to investigate how the filter should be translated for use in PubMed.

Although the SSS filter was only validated against studies published in core clinical journals, it is probably also useful for searching other journals. This will also require further research. Two interesting sources of information that do not belong to the core set of clinical journals are the leading women's health journals *Gender Medicine *and the *Journal of Women's Health *(formerly also known as *Journal of Women's Health and Gender-Based Medicine*). We performed an extra search on Alzheimer's disease and on asthma in the 2007 and 2008 issues of these journals and found two publications reporting sex-specific evidence relevant to clinical practice [39,40]. This suggests that it may be worthwhile to extend searches with the SSS filter to these leading women's health journals.

The SSS filter had a different aim than the previously developed filter by Montgomery and Sherif [26]. Yet, many of the search terms that were included in the two filters were similar. This may explain why their performance was rather similar.

Half of the papers of the development set and one-third of the papers of the reference set could have been identified through a search with sex-specific MeSH only. This indicates that it is not sufficient to rely on the index terms in MEDLINE (MeSH) to seek sex-specific research reports.

The SSS filter is able to identify articles that report sex-specific evidence by screening the title, the abstract and the MeSH that are assigned to them. It should be acknowledged, however, that many authors who report sex-specific data may not mention this in the title or the abstract of their article. For that reason the SSS filter may only be able to retrieve a subset of all available information on the topic. Indeed, this is a common restriction of all search filters.

## Conclusion

We developed a search filter for the retrieval of high quality sex-specific clinical research data in MEDLINE. The recall is high and it has a good precision rate. Although the filter has been developed for guideline organizations, it has also potential relevance to a much wider spectrum of users of clinical evidence, e.g. researchers, medical educators and practitioners. The utility of the filter may vary for different disease categories, journals or research designs. This underlines the need for further evaluation.

## Competing interests

The authors declare that they have no competing interests.

## Authors' contributions

JH initiated the project. CM, JH and RD drew up the design. CM selected the papers for filter development. RD developed the filter. CM selected the papers used to evaluate the filter, designed the criteria for review and critically reviewed the papers with the assistance of JH. JH and CM wrote the paper. RD provided comments on the final version of the paper.

## Pre-publication history

The pre-publication history for this paper can be accessed here:

http://www.biomedcentral.com/1471-2288/9/25/prepub
